# Supplementary material for: Enhancing bacteriophage therapeutics through in situ production and release of heterologous antimicrobial effectors
Source: Nat Commun. 2023 Jul 20;14:4337. doi: 10.1038/s41467-023-39612-0 (PMC10359290; doi:10.1038/s41467-023-39612-0)
Supplement: Supplementary file 1 — Supplementary Information [file 41467_2023_39612_MOESM1_ESM.pdf]

Enhancing bacteriophage therapeutics through in situ production and release of heterologous antimicrobial effectors

Supplementary Material

Supplementary Figures

Supplementary Fig. 1. Analysis of UTI incidents within the Zurich Uropathogen Collection.

Supplementary Fig. 2. Phage resistance development upon treatment of UTI isolates with wildtype E2 and K1.

Supplementary Fig. 3. Reporter phage-based urinalysis of patient urine samples with phage and colicin E7 susceptibility screening of isolated, patient-derived *E. coli* strains.

Supplementary Fig. 4. Further in vitro analysis of HEPT activity against clinical *E. coli* strains of different phage- and ColE7-sensitivity.

Supplementary Tables

Supplementary Table 1. Phages used in the present study and their propagation hosts.

Supplementary Table 2. Peptidoglycan hydrolase (EC300) payload assessment.

Supplementary Table 3. Primers and templates used for synthetic HEPTs construction.

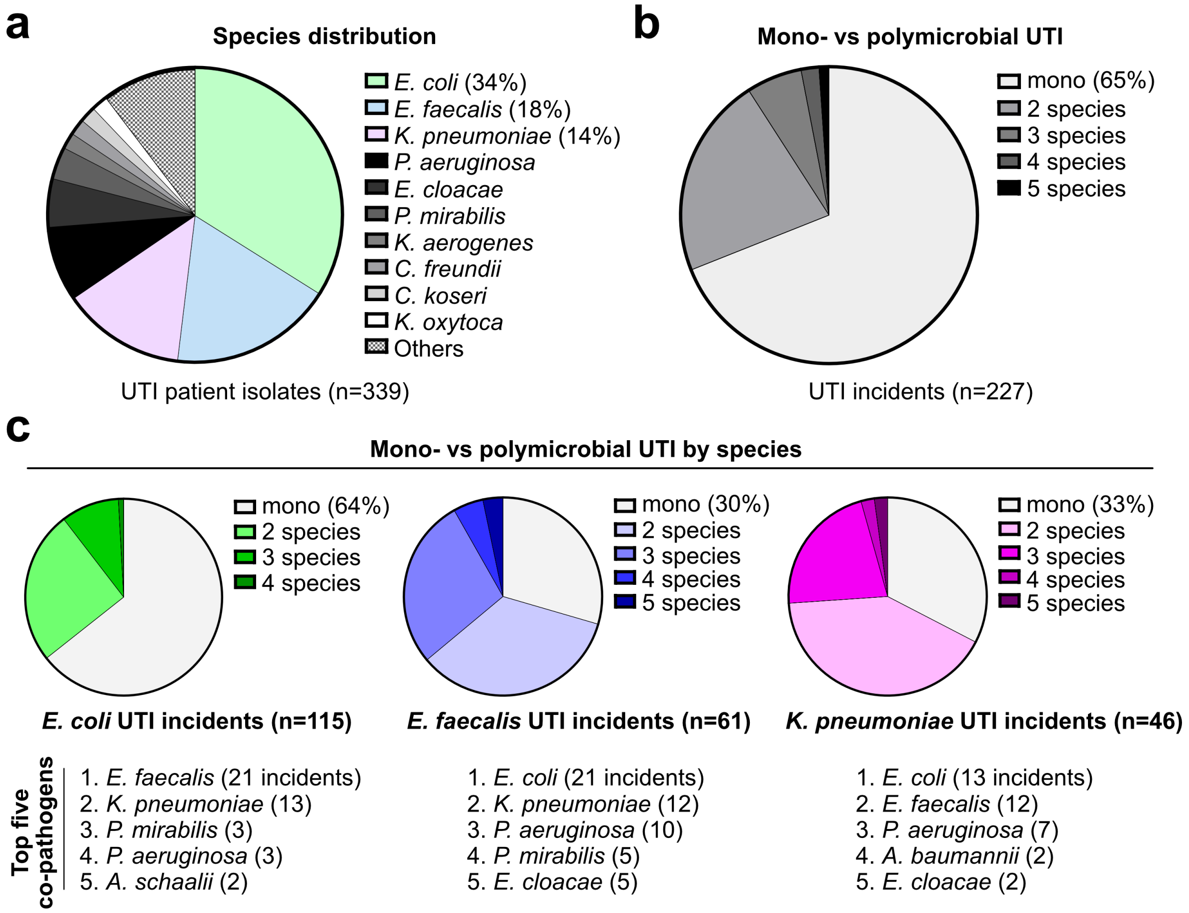

Supplementary Fig. 1. Analysis of UTI incidents within the Zurich Uropathogen Collection. The Zurich Uropathogen Collection[1] comprises 663 isolates from 442 incidents of asymptomatic bacteriuria (n=215) or UTI (n=227). (**a**) The species distribution of 339 isolates acquired from UTI patients was determined and (**b**) the occurrence of mono- vs polymicrobial infections quantified for the corresponding UTI incidents. (**c**) UTI incidents involving the top three uropathogens were analyzed separately to determine the top five co-infecting species and the frequency of mono- and polymicrobial UTIs. Source data are provided as a Source Data file.

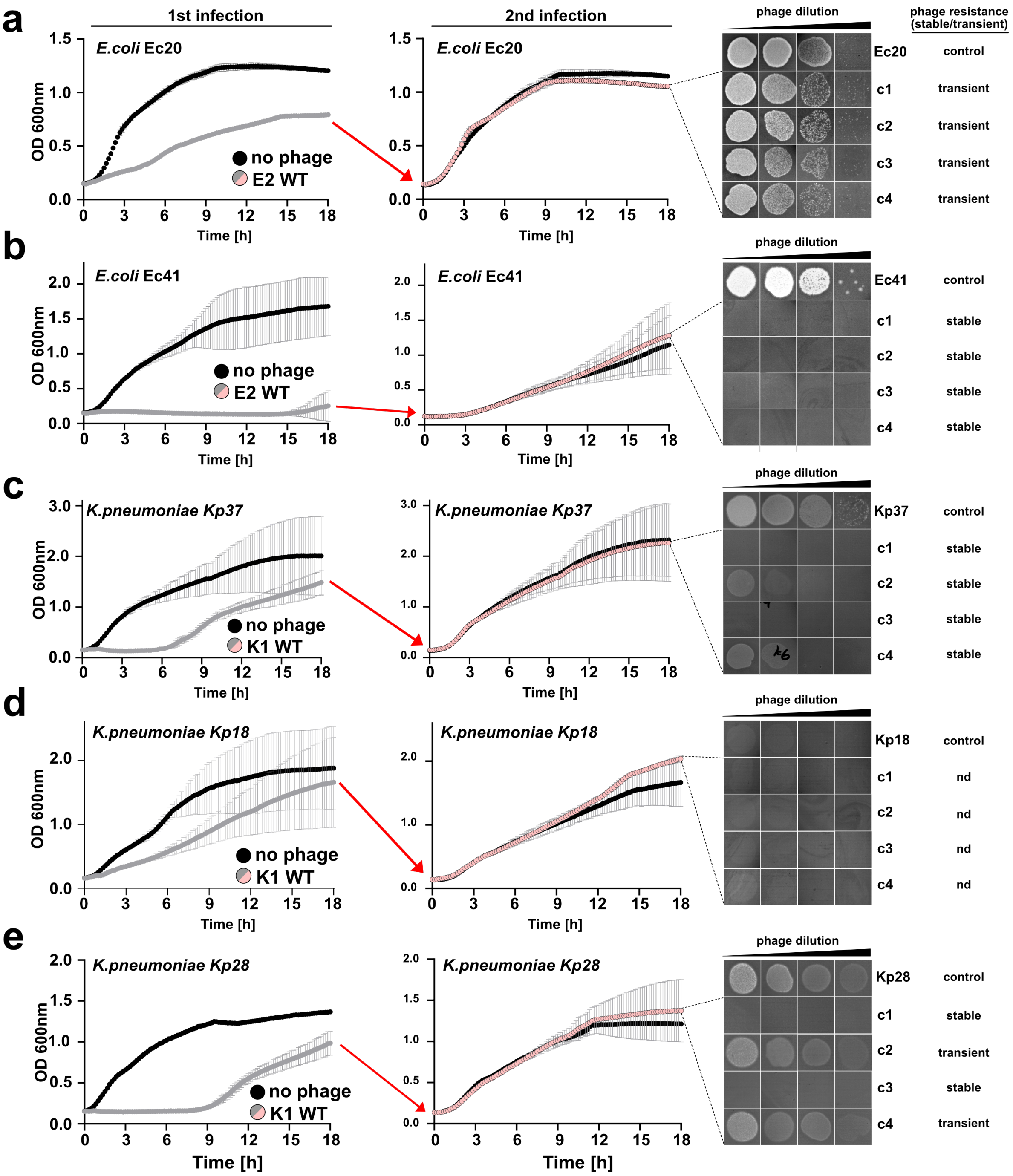

Supplementary Fig. 2. Phage resistance development upon treatment of UTI isolates with wildtype E2 and K1. Phage resistance development was assessed by two consecutive rounds of phage infection of bacterial cultures in SHU medium. Turbidity reduction assays were performed for *E. coli* isolates Ec20 **(a)** and Ec41 **(b)** or *K. pneumoniae* isolates Kp37 **(c)**, Kp18 **(d)** and Kp28 **(e)** infected with 10^8 PFU/mL of phages E2 or K1, respectively, with optical density monitored for 18 h. Phage-exposed cultures were combined (n=3), re-adjusted to OD_{600nm} of 0.1 in SHU and incubated for another 18 h with additional wildtype (WT) phages (pink) or media alone (black). Growth kinetics were compared to non-infected controls. After the second round of infection, individual clonal survivors were isolated, purified via three consecutive rounds of streaking, and assessed for phage susceptibility using spot-on-the-lawn assays. Progenies of bacterial survivors that retained resistance to phage plaquing were considered as stably resistant, whereas those that regained permissiveness to phage plaquing were rendered transiently resistant. Non-infected clones served as positive controls. Turbidity data are technical triplicates shown as mean ± SD. nd = not determined. Source data are provided as a Source Data file.

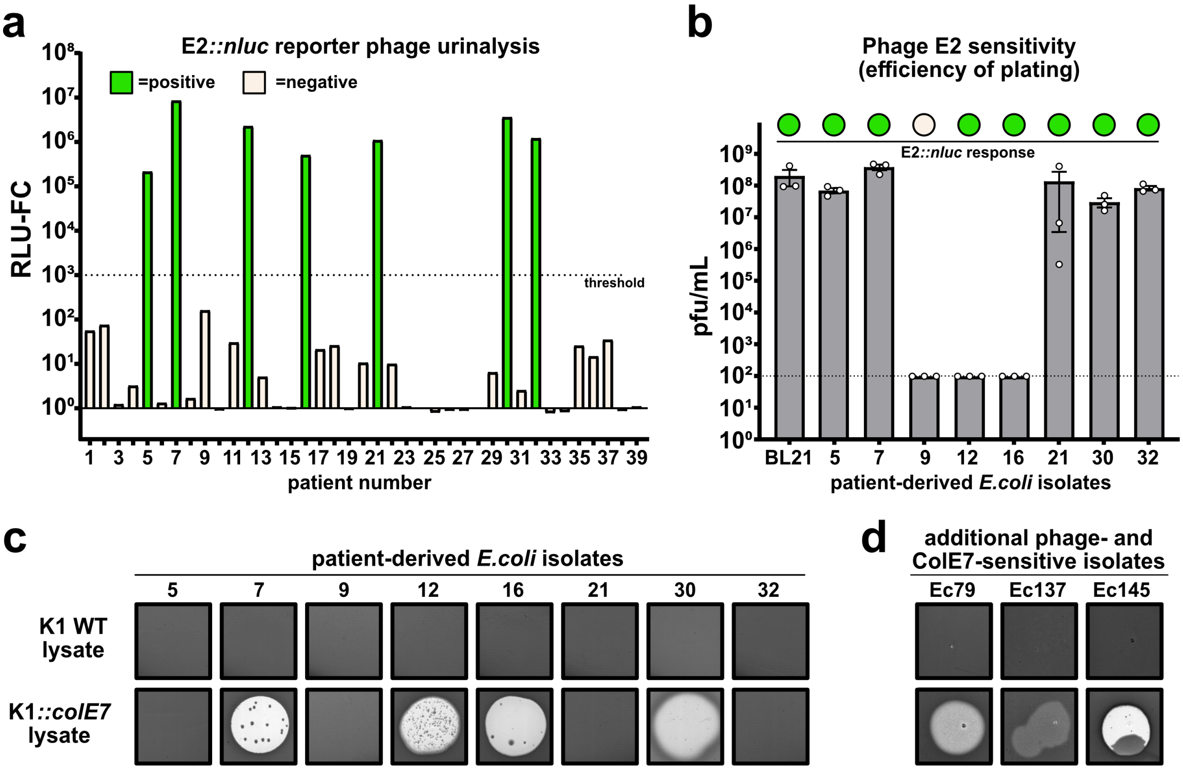

Supplementary Fig. 3. Reporter phage-based urinalysis of patient urine samples with phage and colicin E7 susceptibility screening of isolated, patient-derived *E. coli* strains. (a) Reporter phage urinalysis was performed using 39 fresh patient urine samples from the Balgrist University Hospital, Zurich, Switzerland, as described in **Fig. 4a.** The fold change (compared to phage-only control) in relative light units (RLU-FC) was determined at 3 hours post-infection with urine samples producing values $>10^3$ considered as positive samples, i.e., containing E2-susceptible *E. coli*. Values are derived from single experiment. **(b)** All *E. coli* strains were isolated and purified from patient urine after differential plating, and E2-susceptibility quantified using plaque assays (efficiency of plating). Dotted line indicates the detection limit of plaque assays. Values are derived from three independent experiments and depicted as mean ± SEM. **(c-d)** Colicin E7 effector susceptibility was determined on patient-derived *E. coli* strains from this study (**c**) or from the Zurich Uropathogen Collection[1] (**d**) by spotting K1 WT or K1::*colE7* phage lysates onto growing bacterial lawns of the indicated bacterial isolates (see also **Fig. 1d** for strains Ec82, Ec47, and MVAST0072). Source data are provided as a Source Data file.

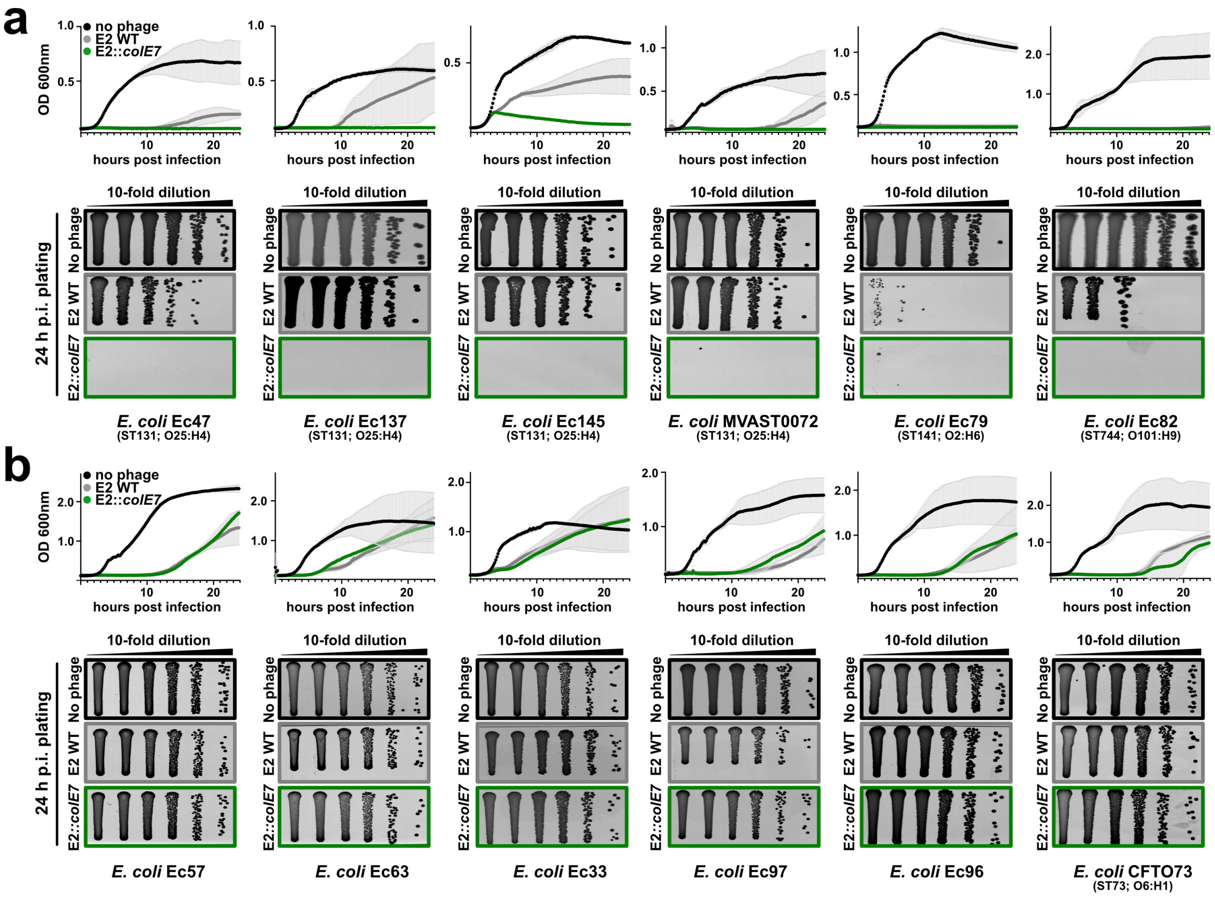

Supplementary Fig. 4. Further in vitro analysis of HEPT activity against clinical *E. coli* strains of different phage- and ColE7-sensitivity. Performance of E2 wildtype (WT) and E2::*colE7* was compared against phage- and ColE7-sensitive strains (**a**) and phage-sensitive but ColE7-resistant strains (**b**) from the Zurich Uropathogen Collection[1] as well as *E. coli* MVAST0072 using 24 h turbidity reduction assays in SHU with endpoint plating. In accordance with data provided in **Fig. 4**, enhanced and sustained killing was observed with E2::*colE7* for all strains showing sensitivity to both phage and ColE7. OD_{600nm} = optical density at 600 nm. Turbidity data are technical triplicates shown as mean ± SD. Source data are provided as a Source Data file.

Supplementary Table 1. Phages used in the present study and their propagation hosts.

Phage	Taxonomic classification (GenBank #)	Genome Size [bp]	Payload Target	Payload Nature	Propagation Host	Source
E2 WT	*Caudoviricetes; Straboviridae; Tevenvirinae; Tequatrovirus* (UTI-E2; OL870316)	166,367	-	-	*E. coli* BL21	[1]
E2::*kvarM*		167,219	*Klebsiella spp.*	colM-like murein synthesis inhibitor	*E. coli* BL21	this study
E2::*colE7*		168,119	*E. coli*	unspecific cytosolic nuclease	*E. coli* BL21 (pIm_immE7)	this study
E2::*ec300*		167,291	*Enterococcus*	chimeric cell wall-hydrolase	*E. coli* BL21	this study
E2::*nluc*		166,904	*E. coli*	Nanoluciferase	*E. coli* BL21	[1]
K1 WT	*Caudoviricetes; Straboviridae; Tevenvirinae; Jiaodavirus* (UTI-K1; OL870318)	170,051	-	-	*K. pneumoniae* KpGe	[1]
K1::*kvarM*		170,903	*Klebsiella spp.*	colM-like murein synthesis inhibitor	*K. pneumoniae* KpGe	this study
K1::*colE6*		171,728	*E. coli*	16s rRNase	*K. pneumoniae* KpGe (pIm_immE6)	this study
K1::*colE7*		171,803	*E. coli*	unspecific cytosolic nuclease	*K. pneumoniae* KpGe (pIm_immE7)	this study
K1::*colM*		170,888	*E. coli*	murein synthesis inhibitor	*K. pneumoniae* KpGe	this study
CM001 WT	*Caudoviricetes; Guernseyvirinae; Kagunavirus* (UTI-CM001; OM810255)	41,222	-	-	*E. coli* Ec20	this study
CM001::*ec300*		42,146	*Enterococcus*	chimeric cell wall-hydrolase	*E. coli* Ec20	this study
EfS3 WT	*Caudoviricetes; Herelleviridae; Brockvirinae; Schiekvirus* (UTI-EfS3; OL870611)	150,393	-	-	*E. faecalis* JH2-2	[1]
EfS3::*colE7*		152,140	*E. coli*	unspecific cytosolic nuclease	*E. faecalis* JH2-2	this study
EfS3::*colM*		151,225	*E. coli*	murein synthesis inhibitor	*E. faecalis* JH2-2	this study
EfS3::*kvarM*		151,240	*Klebsiella spp.*	colM-like murein synthesis inhibitor	*E. faecalis* JH2-2	this study
EfS7 WT	*Caudoviricetes; Saphexavirus* (UTI-EfS7; OL870612)	56,144	-	-	*E. faecalis* Ef57	[1]
EfS7::*colE7*		57,891	*E. coli*	unspecific cytosolic nuclease	*E. faecalis* Ef57	this study
EfS7::*colM*		56,976	*E. coli*	murein synthesis inhibitor	*E. faecalis* Ef57	this study
EfS7::*kvarM*		56,991	*Klebsiella spp.*	colM-like murein synthesis inhibitor	*E. faecalis* Ef57	this study

Supplementary Table 2. Peptidoglycan hydrolase (EC300) payload assessment. 10 µL of phage lysate (10^9-10^{10} PFU/mL) was spotted on bacterial lawns of the following isolates and activity assessed visually after 16 h incubation at 37°C. ++, complete lysis (clear zone); +, moderate lysis (turbid zone); -, no visible activity. Sources: 1, gift from Leo Meile, ETH Zurich, Switzerland; 2, Zurich Uropathogen Collection (2020).

			***E. coli* phages**	
**Species**	**Designation**	**Source**	**E2::*ec300***	**CM001::*ec300***
E. faecalis	JH2-2	1	++	++
E. faecalis	Efs3	2	+	++
E. faecalis	Efs12	2	+	++
E. faecalis	Efs17	2	+	++
E. faecalis	Efs26	2	+	++
E. faecalis	Efs29	2	++	++
E. faecalis	Efs38	2	+	+
E. faecalis	Efs48	2	+	++
E. faecalis	Efs49	2	+	++
E. faecalis	Efs57	2	+	++
E. faecalis	Efs58	2	+	++
E. faecalis	Efs73	2	++	++
E. faecalis	Efs90	2	-	-

Supplementary Table 3. Primers and templates used for synthetic HEPTs construction.

Synthetic HEPTs	Fragment ID	Template	Fragment length (bp)	Primers	Primer Sequence (5'-3')
CM001::*ec300*	CM001 F1	CM001 gDNA	3391	CM001 F1.1 Fw	TCAACGCTTGACAGCCGCA
				CM001 F1.1 Bw	TTACTTGTCCGCGTCGGCG
	CM001_ec300	E/K_ec300 synthetic gene string	974	CM001_ec300 Fw	GGCAATCGCCGACGCGGACAAGTAAAGTACGAGGAGGTAAATATAT
				CM001_ec300 Bw	CCCCTTTGTTTTTACTCCAACCGTATTAAGATTTTTTGGTGATACC
	CM001 F1.2	CM001 gDNA	6433	CM001 F1.2 Fw	TACGGTTGGAGTAAAAACAAAGGG
				CM001 F1.2 Bw	TCGGCCTCAGCTTCGTAATAA
	CM001 F2	CM001 gDNA	10013	CM001 F2 Fw	AAGGATTAAATAATGAAACTTTCTGATTT
				CM001 F2 Bw	TTATTTATCTTCTAGTGCTGCCA
	CM001 F3	CM001 gDNA	11231	CM001 F3 Fw	GTTCTTCATAGAGATTGCCTATC
				CM001 F3 Bw	AGTACAAATCATCAAAGTAAGCA
	CM001 F4	CM001 gDNA	10374	CM001 F4 Fw	ACTATTAACTTTGTCATTGTATAATCCTTGT
				CM001 F4 Bw	ACCCTGCAAATCGTACTGGT
EfS7::*colE7*	EfS7 F1.1	EfS7 gDNA	12126	EfS7 F1.1 Fw	AAAATCCTCTATAAGGCGTCC
				EfS7 F1.1 Bw	ACTTGAGCATCAATAACCCAC
	EfS7_colE7	pEdit_EfS7_colE7	2539	EfS7 colE7 Fw	AAATGACTGATAGCTACGAGTG
				EfS7 colE7 Bw	TTTGCTTCCGTCAGAAGC
	EfS7 F2.1	EfS7 gDNA	10031	EfS7 F2.1 Fw	TTCTCAAAAGACTATGTCTCTAGC
				EfS7 F2.1 Bw	AACCCTTGCAAACTTCTTACC
	EfS7 F3	EfS7 gDNA	11293	EfS7 F3 Fw	ACTTGCCTCCTGAAACTTGG
				EfS7 F3 Bw	TCCTTTAGTGTCATTATCAGTGC
	EfS7 F4	EfS7 gDNA	11182	EfS7 F4 Fw	CGACAACATCATCATAGGCACT
				EfS7 F4 Bw	GTTCATCATAACCTACGTGACC
	EfS7 F4	EfS7 gDNA	10961	EfS7 F5 Fw	CATAAATCCATTCTAAGAGGTCACG
				EfS7 F5 Bw	CTTGTCGGGAAGTGTGTC

Supplementary References

1. Meile, S. et al. Engineered reporter phages for detection of Escherichia coli, Enterococcus, and Klebsiella in urine. *Nature Communications* https://doi.org/10.1038/s41467-023-39863-x (2023).
