# Supplementary material for: Enhancing bacteriophage therapeutics through in situ production and release of heterologous antimicrobial effectors
Source: Nat Commun. 2023 Jul 20;14:4337. doi: 10.1038/s41467-023-39612-0 (PMC10359290; doi:10.1038/s41467-023-39612-0)
Supplement: Supplementary file 3 — Description of Additional Supplementary Files [file 41467_2023_39612_MOESM3_ESM.pdf]

Description of Additional Supplementary Files

Supplementary Data 1. Bacteriocin payload assessment.

Supplementary Data 2. Plasmids and primers used for phage engineering.

Supplementary Data 3. Synthetic DNA strings used for pSelect and pEdit plasmid generation.
