# Supplementary material for: Enhancing bacteriophage therapeutics through in situ production and release of heterologous antimicrobial effectors
Source: Nat Commun. 2023 Jul 20;14:4337. doi: 10.1038/s41467-023-39612-0 (PMC10359290; doi:10.1038/s41467-023-39612-0)
Supplement: Supplementary file 7 — Reporting Summary [file 41467_2023_39612_MOESM7_ESM.pdf]

nature portfolio

Corresponding author(s): Matthew Dunne

Last updated by author(s): Jun 2, 2023

Reporting Summary

Nature Portfolio wishes to improve the reproducibility of the work that we publish. This form provides structure for consistency and transparency in reporting. For further information on Nature Portfolio policies, see our Editorial Policies and the Editorial Policy Checklist.

Statistics

For all statistical analyses, confirm that the following items are present in the figure legend, table legend, main text, or Methods section.

n/a	Confirmed	
☐	☒	The exact sample size (*n*) for each experimental group/condition, given as a discrete number and unit of measurement
☐	☒	A statement on whether measurements were taken from distinct samples or whether the same sample was measured repeatedly
☒	☐	The statistical test(s) used AND whether they are one- or two-sided<br>*Only common tests should be described solely by name; describe more complex techniques in the Methods section.*
☒	☐	A description of all covariates tested
☒	☐	A description of any assumptions or corrections, such as tests of normality and adjustment for multiple comparisons
☐	☒	A full description of the statistical parameters including central tendency (e.g. means) or other basic estimates (e.g. regression coefficient) AND variation (e.g. standard deviation) or associated estimates of uncertainty (e.g. confidence intervals)
☒	☐	For null hypothesis testing, the test statistic (e.g. *F*, *t*, *r*) with confidence intervals, effect sizes, degrees of freedom and *P* value noted<br>*Give P values as exact values whenever suitable.*
☒	☐	For Bayesian analysis, information on the choice of priors and Markov chain Monte Carlo settings
☒	☐	For hierarchical and complex designs, identification of the appropriate level for tests and full reporting of outcomes
☒	☐	Estimates of effect sizes (e.g. Cohen's *d*, Pearson's *r*), indicating how they were calculated

Our web collection on statistics for biologists contains articles on many of the points above.

Software and code

Policy information about availability of computer code

Data collection	GloMax® Navigator Luminometer (Version 3.0, Promega)<br>SPECTROstar Omega (software V5.50, firmware V1.51)<br>SPECTROstar Nano (software V5.70, firmware V1.21).
Data analysis	GraphPad Prism (Version 9)<br>Spectrostar Omega MARS Data Analysis Software (Version 3.42)<br>CLC Genomics Workbench (Version 20)<br>RAST server (https://rast.nmpdr.org/)<br>tRNAscan-SE version 2 (http://lowelab.ucsc.edu/tRNAscan-SE/)<br>MLST and SeroTypeFinder (www.genomicepidemiology.org/)<br>Biorender.com

For manuscripts utilizing custom algorithms or software that are central to the research but not yet described in published literature, software must be made available to editors and reviewers. We strongly encourage code deposition in a community repository (e.g. GitHub). See the Nature Portfolio guidelines for submitting code & software for further information.

Data

Policy information about availability of data

All manuscripts must include a data availability statement. This statement should provide the following information, where applicable:

- Accession codes, unique identifiers, or web links for publicly available datasets
- A description of any restrictions on data availability
- For clinical datasets or third party data, please ensure that the statement adheres to our policy

As provided in the main text, "The phage CM001 genome is available from the GenBank database (UTI-CM001, OM810255) alongside previously sequenced genomes of phage E2 (OL870316), K1 (OL870318), EfS3 (OL870611), and EfS7 (OL870612). Original data used to analyze UTI incidences (Fig. S1) were sourced from Meile et al. (19). All other source data are provided with this paper."

Hyperlinks are provided in the main text for all GenBank entries.

Human research participants

Policy information about studies involving human research participants and Sex and Gender in Research.

Reporting on sex and gender	Data presented in Figure 4 of this study uses urine samples collected from patients of female and male sex (n=39) who were receiving treatment at the tertiary Neuro-Urology Department (Balgrist University Hospital, University of Zurich, Switzerland). Our study tested the antimicrobial activity of engineered bacteriophages (HEPTs) versus non-engineered parental phages direct in patient-derived urine, which was performed irrespective of sex and/or gender. As such, both categories were not importance to our research and were not reported.
Population characteristics	We do not analyze covariate population characteristics in this study. Patients of either sex, ≥18 years old, and who were receiving treatment at the tertiary Neuro-Urology department (Balgrist University Hospital, University of Zurich, Switzerland) were considered eligible.
Recruitment	If a urine culture was indicated as part of a patient's treatment (i.e., for prophylactic reasons prior to invasive diagnostics and/or surgery or due to UTI symptoms) then the patient was asked to provide written informed consent to reuse health-related personal data and their biological material (urine sample) for research purposes. An additional urine sample was then collected and provided to researchers at ETH Zurich to assess reporter phage-based detection and HEPT antimicrobial activity as outlined in Figure 4. Therefore, no self-selection or other bias is likely present.
Ethics oversight	As described in the main text, for the collection of patient urine: "All patients gave a general written informed consent, in line with the local ethics committee (Kantonale Ethikkommission Zurich, Switzerland), agreeing for further use of health-related personal data and biological material for research purposes. The study was performed in accordance with the World Medical Association Declaration of Helsinki (41) and conformed with the International Conference on Harmonisation (ICH) Good Clinical Practice (GCP) Guidelines (E6) and the International OrganizationAll patients gave a general written informed consent, in line with the local ethics committee (Kantonale Ethikkommission Zurich, Switzerland), agreeing for further use of health-related personal data and biological material for research purposes. The study was performed in accordance with the World Medical Association Declaration of Helsinki (41) and conformed with the International Conference on Harmonisation (ICH) Good Clinical Practice (GCP) Guidelines (E6) and the International Organization for Standardization (ISO, 14,155). for Standardization (ISO, 14,155)."

Note that full information on the approval of the study protocol must also be provided in the manuscript.

Field-specific reporting

Please select the one below that is the best fit for your research. If you are not sure, read the appropriate sections before making your selection.

☒ Life sciences ☐ Behavioural & social sciences ☐ Ecological, evolutionary & environmental sciences

For a reference copy of the document with all sections, see nature.com/documents/nr-reporting-summary-flat.pdf

Life sciences study design

All studies must disclose on these points even when the disclosure is negative.

Sample size	No statistical method was performed to predetermine sample sizes for our experiments. Instead, the sample sizes listed below were chosen based on practical considerations, previous similar studies in the field, and the specific objectives of each experiment.<br><br>Samples sizes and replicates (biological or technical) are listed below for all experiments performed in this study:<br><br>Fig. 1. Representative spot-on-the-lawn images are shown for 7 different strains of a single species (E. coli or Klebsiella) for each engineered phage (HEPT) to measure heterologous effector activity. 7 representative images taken from an analysis of 56 E. coli and 59 Klebsiella spp.

strains as listed in Supp. Table 2 & 3.
Fig. 2 c, e, g: for each, two independent experiments were performed as technical triplicates using different strain combinations (two strains per experiment; a producer (phage target) and a recipient (payload target)) to demonstrate the activity of HEPTs in different polymicrobial combinations. Data were reported as mean ± standard deviation.
Fig. 2 d, f, h: strain combinations selected from one of the independent experiments shown in c, e, or g were also analyzed by measuring CFU/mL vs. time as biological triplicates (mean +/- Standard deviation shown).
Fig. 3 c, d: for each, two independent experiments were performed using monocultures of different clinical isolates to demonstrate the enhanced bactericidal activity of self-targeting HEPTs. Turbidity reduction assays were performed as technical triplicates and reported as mean ± standard deviation. At indicated time points (10 and/or 18 h post infection), triplicate samples were combined, serially diluted, and plated on LB agar plates.
Fig. 3 e, f: The same strain combination (E. coli Ec41 and K. pneumoniae Kp37) was used to demonstrate improved antimicrobial activity of self-HEPT (e) and cross-HEPT (f) cocktails through two independent experiments. Turbidity reduction assays were performed as technical triplicates and reported as mean ± standard deviation. At measurement endpoint, triplicate samples were combined, serially diluted, and plated on differential coliform agar.
Fig. 4 b urinalysis: As described below for Supp. Fig. 3a, reporter phage (E2::nluc) detection results for the 39 patient-derived urine samples were deduced from bioluminescence assay (biological duplicates). E. coli plating results were concluded from single plating experiment on differential agar.
Fig. 4 b in vitro: As described below for Supp. Fig. 3b, plaque formation results for the 8 patient-derived E. coli isolates were deduced from soft-agar-overlay assay (biological triplicates). ColE7 sensitivity results were concluded from single effector spotting assay.
Fig. 4c: EX VIVO data: Ex vivo time kill assay was performed for the 6 E. coli-positive patient urines as single measurement. Each individual patient urine was treated independently, with its results reported as mean of colony counts calculated from 2-3 countable serial dilutions.
Fig. 4c, IN VIVO data: data is shown as mean +/- SD of technical triplicate data using isolates from the 6 independent experiments reported in the EX VIVO analysis.
Supp. Fig. 2: technical triplicate data shown for turbidity reduction curves for five independent experiments.
Supp. Fig. 3a: Reporter phage urinalysis was performed as biological duplicates independently for 39 fresh patient urine. For each sample, bioluminescence results were background corrected and reported as mean RLU-FC (fold change in relative light unit).
Supp. Fig. 3b: The phage susceptibility of the eight patient-derived E. coli isolates and one positive control (BL21) was quantified using the soft-agar-overlay method. Each individual E. coli isolate was tested independently in biological triplicates, with its efficiency-of-plating (EOP) reported as mean ± standard deviation.
Supp. Fig. 4 a, b: Six different phage- and ColE7-sensitive clinical E. coli isolates (a) were compared with six different phage-sensitive but ColE7-resistant E. coli isolates (b) to demonstrate the enhanced bactericidal activity of E2::colE7 on double sensitive hosts. Each turbidity reduction assay was performed independently as technical triplicates and reported as mean ± standard deviation. At measurement endpoint, triplicate samples were combined, serially diluted, and plated on LB agar plates.
Supp. Table 2 and 3: As described above, Supp. Table 2 shows activity data of individual cross-HEPTs tested against 56 E. coli and 59 Klebsiella spp. strains. In Supp. Table 3, payload activity of E2::ec300 and CM001::ec300 were tested independently using 13 different Enterococcal strains.

Data exclusions

no data was excluded

Replication

A single transmission electron microscopy image of each phage (Fig. 1b) was selected from multiple micrographs taken from a single phage-coated grid as is common practice. All TKAs are biological triplicates excluding the ex vivo urine treatment (performed once as done with fresh urine directly). The field evaluation including bioluminescence-based detection and ex vivo TKA were both performed once due to time and material limitations of working with multiple fresh urine samples (efficiency of plating was performed with isolated strains; Fig. S3b). All TRA (and corresponding endpoint CFU plating) and spot lysis data were reproduced at least two times, with one set of data shown.

Randomization

There was no requirement for randomization as all samples and data points in this study were analyzed and reported equally with no sub-sampling. For instance, the urine samples analyzed in Fig. 4 were simply collected consecutively throughout sampling days, without any pre-selection based on age, sex, gender, or treatment plan. In the lab, these samples were all tested and reported the same way with no sample exclusion.

Blinding

Blinding was not required in this study as all samples and data points were tested and analyzed equally with no sub-sampling. Furthermore, all urine samples were tested blind to any prior microbiological identification as results from standard-of-care diagnostics were not available at the time of conducting the reporter phage analysis or the EX VIVO time kill assay analysis (Fig. 4c).

Reporting for specific materials, systems and methods

We require information from authors about some types of materials, experimental systems and methods used in many studies. Here, indicate whether each material, system or method listed is relevant to your study. If you are not sure if a list item applies to your research, read the appropriate section before selecting a response.

Materials & experimental systems

n/a	Involved in the study
☒	☐ Antibodies
☒	☐ Eukaryotic cell lines
☒	☐ Palaeontology and archaeology
☒	☐ Animals and other organisms
☒	☐ Clinical data
☒	☐ Dual use research of concern

Methods

n/a	Involved in the study
☒	☐ ChIP-seq
☒	☐ Flow cytometry
☒	☐ MRI-based neuroimaging
